# Cell Biological Techniques and Cell-Biomaterial Interactions

**DOI:** 10.3390/cells9092094

**Published:** 2020-09-14

**Authors:** Yunqing Kang

**Affiliations:** 1 Department of Ocean and Mechanical Engineering, College of Computer Science and Engineering, Florida Atlantic University, Boca Raton, FL 33431, USA; kangy@fau.edu; 2 Department of Biomedical Science, College of Medicine, Florida Atlantic University, Boca Raton, FL 33431, USA; 3 Faculty of Integrative Biology PhD program, College of Science, Florida Atlantic University, Boca Raton, FL 33431, USA

**Keywords:** cell-biomaterial interactions, cell biological techniques, tissue engineering

## Abstract

Biomaterials play a key role in modern tissue engineering and regenerative medicine. They are expected to take over the function of a damaged tissue in the long term, trigger the self-healing potential of the body, and biodegrade at an appropriate rate. To meet these requirements, it is imperative to understand the cell-biomaterial interactions and develop new cell biotechnologies. The collection of this Special Issue brings together a number of studies portraying the underlying mechanisms of cell-biomaterial interactions.

Biomaterial-based technologies and tissue engineering strategies have the promising potential to dramatically impact regenerative medicine [1]. The development of regenerative medicine necessarily needs the involvements of tissue-engineered biomaterials. To effectively regulate cell behavior and direct cell differentiation for tissue regeneration, the understanding of cell-biomaterial interactions is crucial [2]. More studies on the underlying mechanisms of cell-biomaterial interactions may promote the success in translating tissue-engineered biomaterials into clinical applications [3]. The effect of biomaterial properties on cell behavior is profound, and cells recognize and interact with biomaterials in different forms at different levels [4]. Therefore, it is imperative to fully understand the complex interactions between cells and biomaterials in space and in time. To this end, we opened a Special Issue to discuss this topic.

This Special Issue includes five articles to demonstrate several aspects of cell-biomaterial interaction research, cellular engineering techniques, and the development of new cell biological techniques for tissue regeneration.

Smart nanoparticle-based drug delivery systems offer new, wide, functional platforms to efficiently deliver drug molecules to diseased tissues in the body [5]. However, despite the remarkable developments of nanoparticle biotechnologies, their efficient use in nanomedicine is limited due to the barrier of the blood system [6]. Blood systems are the first barrier that nanoparticles need to go through before reaching the targeted tissue when they are administered intravenously [7]. The blood systems contain blood cells, endothelial cells, and proteins. Their interactions with nanoparticles determine the fate of nanoparticles. Understanding the blood-nanoparticles interactions is crucial to successfully design and develop a new smart nanoparticle-based drug delivery system. To this end, in this Special Issue Viness Pillay et al. reviewed blood cell-nanoparticle interactions and hemostasis [8]. In the paper, they highlighted the function of three cellular components of blood, namely erythrocytes, platelets and leukocytes, in hemostasis, and the potential deleterious effects that nanoparticles can have on these cells. At the same time, they provided insightful views about some of the complex mechanisms involved in nanoparticle-blood cell interactions. Based on these mechanisms, the techniques to engineer the surface of nanoparticles were reviewed. These techniques include designing the surface charge, geometry, and porosity of nanoparticles and modifying the surface properties of nanoparticles with specific polymers or functional groups. It is clear that an understanding of blood-nanoparticles interactions is imperative and also necessary to develop new cell biological techniques to bio-functionalize nanoparticles for their efficient use in medicine.

Osteoarthritis (OA) is a common chronic joint disease characterized by pain, deformity, instability, and reduction in motion and function [9]. Although there is a current lack of an effective treatment for OA, many new strategies have been put forward. Carola Cavallo et al. reviewed these new strategies including stem cell-based therapy, gene therapy, and growth factor/3D printing scaffolding option [10]. Although currently these regenerative strategies still have many challenges in completely regenerating dysfunctional cartilages, new opportunities are appearing and new functional biomaterials are being developed to advance the treatment, for example, injectable hydrogel that can modulate the inflammation of the joint. Nathaniel S. Hwang et al. reviewed this aspect function of inflammation-modulating hydrogels for osteoarthritis cartilage tissue engineering [11]. Immunomodulatory biomaterials have been applied in many fields not just OA treatment. In the treatment of OA, injectable/adhesive hydrogels carrying anti-inflammatory drugs, proteins, genes, or cells have inherent immunomodulatory function through regulating immune cell polarization and activity to manage the inflammation of OA. Such immunomodulatory tissue engineering solutions will bring new potential to OA treatment.

Typical treatment for chronic wounds includes application of autografts, allografts, topical delivery of antioxidant, anti-inflammatory, and antibacterial agents [12]. However, these therapeutic applications have limitations, including deficiency in donor skin graft supplies and immuno-rejection of allografts and xenografts. To overcome these drawbacks, advanced therapies including biomaterial-based artificial skin grafts, with the topical delivery of mesenchymal stem cells to reduce inflammation and accelerate the healing process, have been developed [13]. Ekaterina A. Vorotelyak et al. reviewed this topic in this issue [14]. They reviewed the role of hair follicle mesenchyme cells in skin repair and discussed the criteria and effectiveness of skin substitutes in skin reconstruction.

In this Special Issue, a research article was included [15]. Maribel Vazquez’s group utilized a microfluidic technique to examine the migratory responses of retinal progenitor cells upon extracellular substrates to exogenous chemotactic signaling. Their study results show that retinal cluster size and composition influenced retinal progenitor cells clustering upon extracellular matrix. This study highlighted the significance of examining collective cell-biomaterial interactions on biomaterials for retinal regenerative therapies.

Cell-biomaterial interaction research is a fundamental research field with immense challenges but understanding the underlying mechanisms of cell-biomaterial interactions has tremendous potential to revolutionize cell biological and bioengineering techniques for enhancing cell-based and biomaterial-based tissue regenerative medicine.

Finally, we would like to take this opportunity to express our gratitude to all of the authors who contributed to this Special Issue. We also express our appreciation to all the reviewers who provided thorough and timely reviews to ensure the quality of this Special Issue.
